# Cyclic Alternating Pattern Is Associated with Cerebral Hemodynamic Variation: A Near-Infrared Spectroscopy Study of Sleep in Healthy Humans

**DOI:** 10.1371/journal.pone.0046899

**Published:** 2012-10-10

**Authors:** Tiina Näsi, Jaakko Virtanen, Jussi Toppila, Tapani Salmi, Risto J. Ilmoniemi

**Affiliations:** 1 Department of Biomedical Engineering and Computational Science (BECS), Aalto University School of Science, Espoo, Finland; 2 BioMag Laboratory, HUS Medical Imaging Center, Helsinki University Central Hospital, Helsinki, Finland; 3 Department of Clinical Neurophysiology, HUS Medical Imaging Center, Helsinki University Central Hospital, Helsinki, Finland; 4 Department of Neurological Sciences, University of Helsinki, Helsinki, Finland; Tokyo Metropolitan Institute of Medical Science, Japan

## Abstract

The cyclic alternating pattern (CAP), that is, cyclic variation of brain activity within non-REM sleep stages, is related to sleep instability and preservation, as well as consolidation of learning. Unlike the well-known electrical activity of CAP, its cerebral hemodynamic counterpart has not been assessed in healthy subjects so far. We recorded scalp and cortical hemodynamics with near-infrared spectroscopy on the forehead and systemic hemodynamics (heart rate and amplitude of the photoplethysmograph) with a finger pulse oximeter during 23 nights in 11 subjects. Electrical CAP activity was recorded with a polysomnogram. CAP was related to changes in scalp, cortical, and systemic hemodynamic signals that resembled the ones seen in arousal. Due to their repetitive nature, CAP sequences manifested as low- and very-low-frequency oscillations in the hemodynamic signals. The subtype A3+B showed the strongest hemodynamic changes. A transient hypoxia occurred during CAP cycles, suggesting that an increased CAP rate, especially with the subtype A3+B, which may result from diseases or fragmented sleep, might have an adverse effect on the cerebral vasculature.

## Introduction

Traditional sleep scoring (rapid-eye-movement (REM) sleep and non-REM stages S1–4 or N1–3) does not capture all important aspects of sleep [1], [2]. The non-REM stages exhibit a microstructure that is related to consolidation of learning and neurocognitive performance [3], [4]. Moreover, its increased prevalence has been observed in connection with reduced sleep quality either due to natural tendency or diseases, e.g., insomnia, restless leg syndrome, and sleep apnea syndrome [5]–[7]. The microstructure is expressed as recurring events of electrical brain activity that form the so-called cyclic alternating pattern (CAP). In the electroencephalogram (EEG), it shows as recurring transient alpha, clusters of vertex waves, K-complexes, or transient high-amplitude delta or mixed-frequency patterns. It is related to sleep instability and preservation, and is observed even in coma [7], underscoring its fundamental role during states of reduced vigilance.

CAP has been studied for over a decade with EEG, but its cerebral hemodynamic counterpart has not been assessed in healthy subjects so far (hemodynamic changes during CAP have been investigated in three patients with restless legs syndrome [5] and closely related arousals have been studied with pulsed Doppler ultrasonography and functional magnetic resonance imaging (fMRI) [8], [9]). Systemic hemodynamic changes due to autonomic nervous system activation have been reported during CAP [10], [11]. One of the reasons why cerebral hemodynamics during CAP has not been studied so far may be the fast nature of the CAP events, in the order of seconds, which hinders the use of positron emission tomography (PET) in recording its temporal characteristics. On the other hand, the noisy environment, movement restrictions, and difficulty of recording the polysomnogram in fMRI complicate its applicability for sleep recordings. In contrast to PET and fMRI, near-infrared spectroscopy (NIRS) is well-suited for all-night measurements of cerebral hemodynamics: it can be recorded at bedside, it tolerates slight movements, it is relatively comfortable, and its temporal resolution is sufficient for recording CAP events. It can measure scalp and cortical oxy- and deoxyhemoglobin concentration changes (Δ[HbO_2_] and Δ[HbR]) in sleep-stage transitions, as well as characterize their slow oscillatory behavior in separate sleep stages [12], [13]. In addition, it can be applied to measure REM-sleep-related brain activation [14].

Investigating cerebral hemodynamic regulation during normal cyclic sleep discontinuity and spontaneous arousals is the basis for understanding the nature of pathophysiological hemodynamic mechanisms associated with sleep fragmentation disorders, such as sleep apnea, restless legs, insomnia, and pain syndromes [7]. As these disorders cause repetitive discontinuities of sleep, their hemodynamic mechanisms may be related to the increased prevalence of cardiovascular diseases [15]. Moreover, understanding differences and similarities between the normal and pathophysiological mechanisms will advance developing better treatment and preventing complications of these common sleep disorders. In this study, we examine CAP-related scalp, cortical and systemic hemodynamics in healthy subjects with NIRS and pulse oximetry. In particular, we focus on investigating differences between three subtypes of CAP. In addition, we discuss the possible relation of CAP to very-low- (VLF, 0.003–0.04 Hz) and low-frequency (LF, 0.04–0.15 Hz) hemodynamic oscillations [13], which constitute a considerable proportion of physiological variation in NIRS signals [16], [17] and have been attributed in awake subjects to many underlying factors, such as neuronal activity [18], [19], vasomotion [16], and autonomic control of the systemic circulation [20].

## Materials and Methods

### Ethics Statement

All subjects gave a written informed consent prior to participating in the study. The study was accepted by the Ethics Committee of Helsinki University Central Hospital and was in compliance with the Declaration of Helsinki.

### Subjects and Data Acquisition

NIRS, polysomnogram, and pulse oximeter data of 11 healthy subjects (8 men, 3 women, mean age 26 years, range 21–32) during 23 nights, previously analyzed for sleep-stage transitions and spontaneous hemodynamic oscillations [13], were rescored for CAPs by a neurophysiologist. Ten of the subjects were recorded on at least two nights. One recording was done without the pulse oximeter. Originally 13 subjects participated in the study, but the data of one subject were excluded because of detachment of the NIRS probe soon after falling asleep on both measurement nights; the data of another subject were excluded because of evidence of sleep apnea in the pulse oximeter recording.

Frequency-domain NIRS data were recorded on the right side of the forehead. The optode grid comprised three channels with source-to-detector separations of 1, 4, and 5 cm [13], [21]. Attenuation of the light modulation amplitude was converted to Δ[HbO_2_] and Δ[HbR] with the modified Beer–Lambert law; the differential path length factor was estimated from the phase of the optical signals [22]. Data were sampled at 10 Hz, corrected for large discontinuities related to motion artifacts based on accelerometer measurement [23], and low-pass filtered to remove heartbeat and high-frequency noise (Chebyshev type II, zero-phase filtering, −3 dB cutoff: 0.4 Hz). The whole-night data were not detrended. The 4- and 5-cm signals behaved similarly but the 5-cm signal was visibly much noisier due to lower light intensity at the detector. Thus, only data from the 4-cm channel are presented in this study.

The beat-to-beat amplitude of photoplethysmograph (PPGamp) and the beat-to-beat heart rate were obtained from the finger pulse oximeter recording with the sampling rate of 1 Hz, and the arterial oxygen saturation (SpO_2_) with the sampling rate of 0.1 Hz. PPGamp represents the amount of blood pulsating in the finger tip. It generally decreases with vasoconstriction and increases with vasodilation [24], and is negatively correlated with blood pressure [25].

### CAP and Sleep Scoring

The polysomnogram, consisting of EEG (channels C4–A1, O2–A1, C3–A2, and O1–A2), left and right electrooculogram, and chin electromyogram, was scored by a neurophysiologist into sleep stages S1, S2, S3, S4, REM, wake, and movement time in 30-s epochs according to the Rechtschaffen–Kales rules [1]. For data analysis, the sleep stages S1 and S2 were combined into light sleep (LS), and sleep stages S3 and S4 into slow-wave sleep (SWS). He also marked manually CAP phases according to the rules published in Sleep Medicine [6]. Briefly, the rules were:

1. CAP does not appear during REM sleep or wakefulness.
2. Phases A and B alternate in a sequence.
3. Phase A consists of transient alpha, clusters of vertex waves, K-complexes, or transient high-amplitude delta or a mixed-frequency pattern of at least 133% of the background amplitude in phase B.
4. The duration of phases A and B is between 2 and 60 s.
5. Phases A are divided into subtypes 1–3 according to the ratio of EEG synchrony and desynchrony. Subtype A1 consists of a cluster of high-amplitude activity, while subtypes A2 and A3 contain a transient high-amplitude cluster accompanied by desynchronization (>50% of the time in A3, 20–50% in A2).

During the sleep stage and CAP analysis, the scorer was unaware of the simultaneous phenomena in the NIRS and pulse oximeter signals.

### Data Analysis

Power spectral densities (PSDs) were estimated for Δ[HbR], Δ[HbO_2_], PPGamp, and heart rate to quantify the power of VLF and LF oscillations during CAP sequences (A+B+…+B) and during non-CAP. PSDs were calculated separately for each CAP sequence and each non-CAP period with Welch's method (50% segment overlap; 26-s segment length for NIRS data, 32-s for systemic data; 0.020-Hz frequency resolution for NIRS data, 0.016-Hz for systemic data) after removing the mean of the signal. Over 32-s-long CAP sequences and non-CAP periods were included in the analysis; shorter sequences did not allow estimating the PSD reliably. Also, only CAP sequences with at least three cycles (A+B) were included. A non-CAP period was defined to start 40 s subsequent to the end of a CAP sequence. The power of VLF/LF oscillations was obtained from each PSD as the integral over the VLF and LF ranges (0.003–0.15 Hz). Linear data detrending before the PSD calculation did not affect the results.

To quantify hemodynamic changes during CAP cycles, Δ[HbR], Δ[HbO_2_], PPGamp, and heart rate time series were averaged over all separate CAP cycles belonging to one subtype within the time window −5…40 s with respect to the beginning of the cycle. The baseline of each signal before the cycle was set to zero by subtracting the mean of the signal within the period −5…0 s.

To reduce the contribution of motion artifacts, data scored as movement time by the neurophysiologist were not included in the analysis. All sequences and cycles were also screened for artifacts before PSD calculation and averaging as follows: if the difference between the maximum and minimum value within the sequence or cycle exceeded four times the median over all sequences or cycles, the sequence or cycle was not included in the analysis. All NIRS channels and both Δ[HbO_2_] and Δ[HbR] were rejected if either one of the NIRS signals exceeded the threshold in one channel. This procedure led to the rejection of 8–12% of CAP sequences and non-CAP periods in the PSD analyses and 0–3% of CAP cycles in the averaging. These rejection rates were considered satisfactory to exclude data contaminated by artifacts.

The sampling rate of SpO_2_ was too low to show time courses related to CAP cycles. To evaluate the presence of apnea during the cycles, SpO_2_ values after the beginning of A and after the beginning of B were averaged over separate cycles of each subtype.

### Statistical Testing

All averaged time series are shown as mean over cycles ±95% confidence interval calculated with *t*-statistics. The confidence intervals have not been corrected for multiple comparisons.

Kruskal–Wallis one-way analysis of variance (ANOVA) by ranks was applied to test if the durations of phases A1, A2, and A3 differed from each other. This test was selected instead of the conventional ANOVA because box plots and Shapiro–Wilk tests indicated that the durations were not normally distributed. To see which of the durations differed from each other, the post-hoc Wilcoxon rank sum test for equal medians was applied with Bonferroni corrected *p*-values for three combinations.

To test whether the time spent in subtype A3+B differed between SWS and LS, the independent two-sample *z*-test was applied by classifying each epoch of 0.1 s of SWS or LS as belonging to subtype A3+B or not.

To test if the power of VLF/LF oscillations differed between CAP and non-CAP, the power in decibels obtained from the PSD estimates were compared to each other with independent two-sample *t*-test for equal variances. The test was done separately for each signal type, channel, and sleep stage; the *p*-values were corrected with false discovery rate (FDR) control for 12 comparisons [26].

One-way ANOVAs (factor: time; levels: 46 time points) were applied for testing whether the averaged signals showed significant changes [27], [28]. To reduce the number of levels in ANOVA, NIRS signals were down-sampled to 1 Hz by averaging ten consecutive time samples together. The variance of the signal values between time points was compared with the average variance within time points. The null hypothesis was that there are no significant differences in the values between time points. ANOVA was performed separately for each signal type, each channel, and CAP subtype; therefore, *p*-values were adjusted for 18 comparisons with FDR control (3 CAP subtypes for Δ[HbR] and Δ[HbO_2_] in 2 channels, for heart rate, and for PPGamp) [26]. The normality assumption of ANOVA was validated visually with box plots.

Signal peak amplitudes were tested in order to assess whether they depended on the NIRS channel (1 cm, 4 cm), CAP subtype (A1, A2, A3), sleep stage (SWS, LS), and phase A duration (short duration, 2–7 s; intermediate, 7–11 s; long, 11–60 s). To calculate the amplitudes, peak times were estimated from signals averaged separately for each combination of factor levels, except for the sleep stage. Sleep stages were not separated because the rarity of subtypes A2+B and A3+B in SWS (49 cycles of A2+B, 89 cycles of A3+B) reduced the signal-to-noise ratio and sacrificed the reliability of the peak times. Since Δ[HbR] and Δ[HbO_2_] showed two peaks with opposing polarities, the latter, stronger one occurring in phase B was analyzed. The amplitudes were calculated as time averages separately for each cycle around the peak time (±2 s). The amplitudes were tested for factors CAP subtype, phase A duration group, sleep stage, and, in the case of NIRS signals, measurement channel, with three- (heart rate, PPGamp) and four-way (Δ[HbR], Δ[HbO_2_]) ANOVAs. The normality assumption of ANOVA was validated visually with box plots. The *p*-values were corrected with the Bonferroni method for four separate tests. Post-hoc testing for significant interaction (CAP×NIRS channel) was performed with one-way ANOVAs separately for each channel (Bonferroni correction for two channels). Post-hoc testing for significant factors was performed with the Tukey–Kramer method.

## Results

The sleep efficiency (sleep time of the time in bed) was on average 82±16% (mean ± standard deviation (SD) over measurements). The average proportions of SWS, LS, and REM of the total sleep time were 18±7%, 65±10%, and 17±7%, respectively (mean ± SD over measurements).

**Figure 1:**
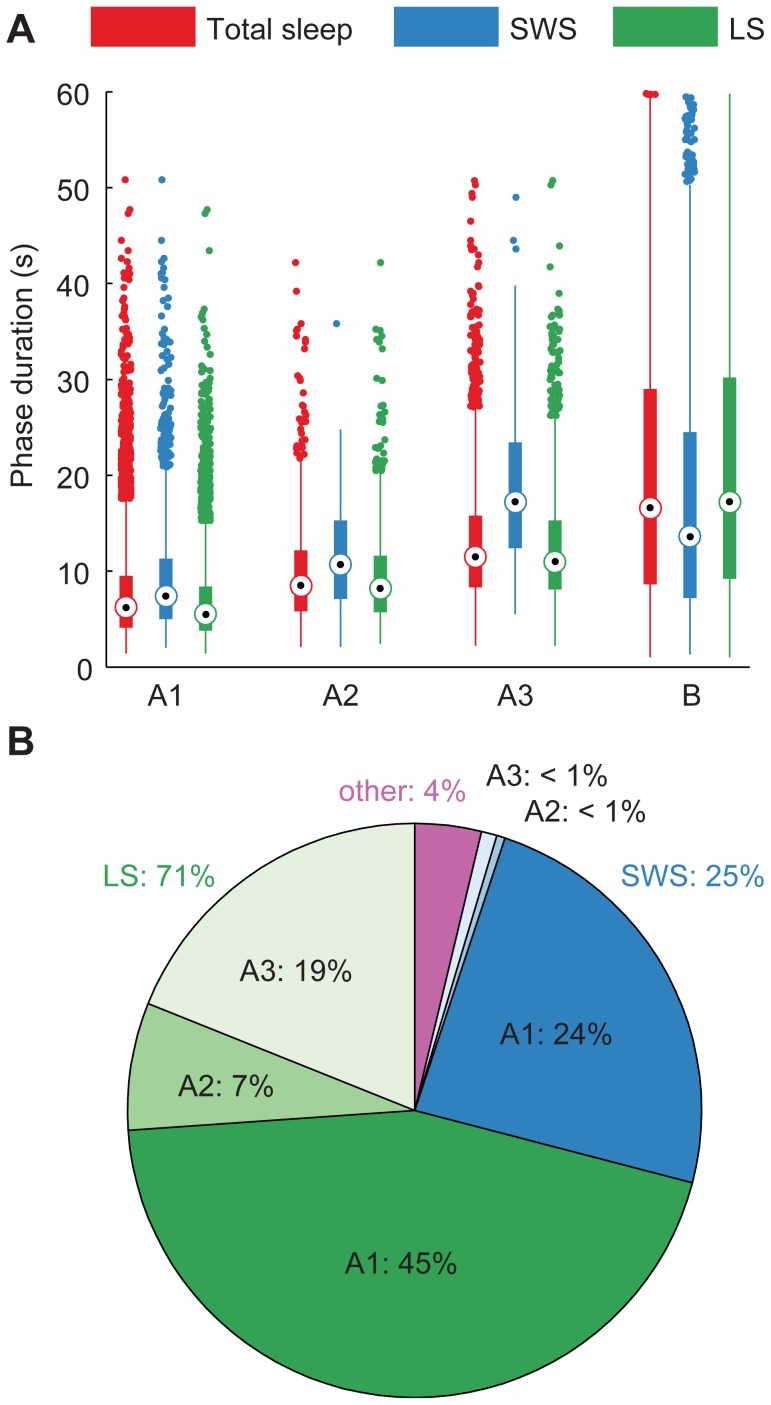
Descriptive statistics of CAP phases. (A) Distribution of phase durations and (B) division of phases into subtypes and sleep stages. Total sleep is marked red, SWS blue, LS green, and other times (movement time or changing sleep stage during the phase) purple. The box plots in (A) show the interquartile range as a box and the median as a circled dot inside the box. If the data includes outliers, i.e. dots outside of the whiskers, the whiskers are 1.5 times the interquartile range. Otherwise, they span the range from the minimum to the maximum value. A3 was on average the longest and A1 the shortest of phases A. Most of the CAP cycles were scored as A1+B and most of them occurred in LS.

CAP sequences were found in the polysomnograms of all subjects. The CAP rate, i.e., percentage of CAP time of the total non-REM time [7], was 64±12% (mean±SD over measurements). A CAP sequence lasted for 252±342 s (mean ± SD over sequences) and comprised on average 9 cycles; a cycle was 29±16 s long (mean ± SD over cycles, used subsequently). Phase A lasted 9±6 s, with subtype A1 having the shortest and A3 the longest duration (Figure 1A). The duration of phase A depended statistically significantly on the subtype, with all subtypes differing significantly from each other in this data set (*p*<10^−41^, Kruskal–Wallis one-way ANOVA and post-hoc Wilcoxon rank sum test with Bonferroni correction). Phase B lasted 20±6 s. Most of the CAP phases were assigned to subtype A1 (71%) and most of them occurred during LS (71%) (Figure 1B). In SWS, only 3% of the total time was assigned to cycles A3+B in this data set, whereas 19% of the time spent in LS belonged to A3+B (*p*<10^−47^ for the difference between SWS and LS, independent two-sample *z*-test).

**Figure 2:**
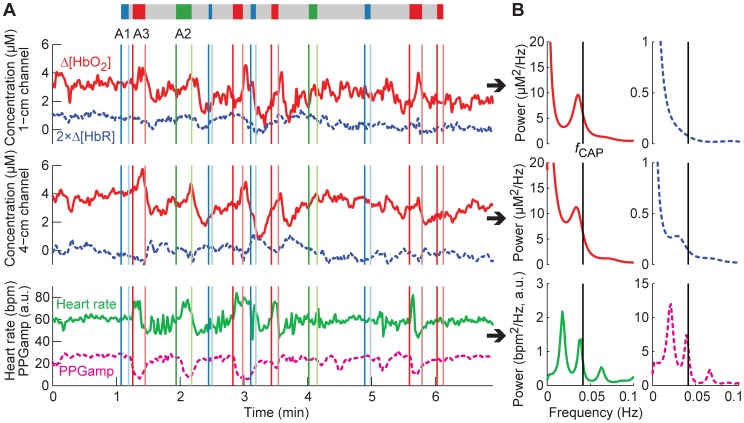
Hemodynamic signals during one CAP sequence. (A) Signals in time domain and (B) corresponding PSD estimates. In (A), Δ[HbR] has been multiplied by two to show its changes more clearly. The CAP sequence is depicted with a colored bar above the graphs (A1: blue, A2: green, A3: red, B: gray). The start times (dark colors) and end times (light colors) of the phases are also marked with vertical lines of the corresponding color. In (B), the PSDs were estimated with the covariance method (model order for NIRS: 200; for systemic data: 50). Phase A3 evokes the strongest hemodynamic changes, but occasionally also the other subtypes produce visible changes. The CAP sequence causes the hemodynamic signals to oscillate roughly at the repetition rate of the cycles (*f*_CAP_).

The CAP cycles produced visible changes in the hemodynamic signals (Figure 2A). In particular during A3, Δ[HbO_2_] increased, PPGamp decreased, and heart rate increased rapidly and thereafter decreased. Opposite changes occurred in Δ[HbO_2_] and PPGamp during the same cycle in B. A1+B and A2+B were accompanied by similar changes, but less regularly.

**Figure 3:**
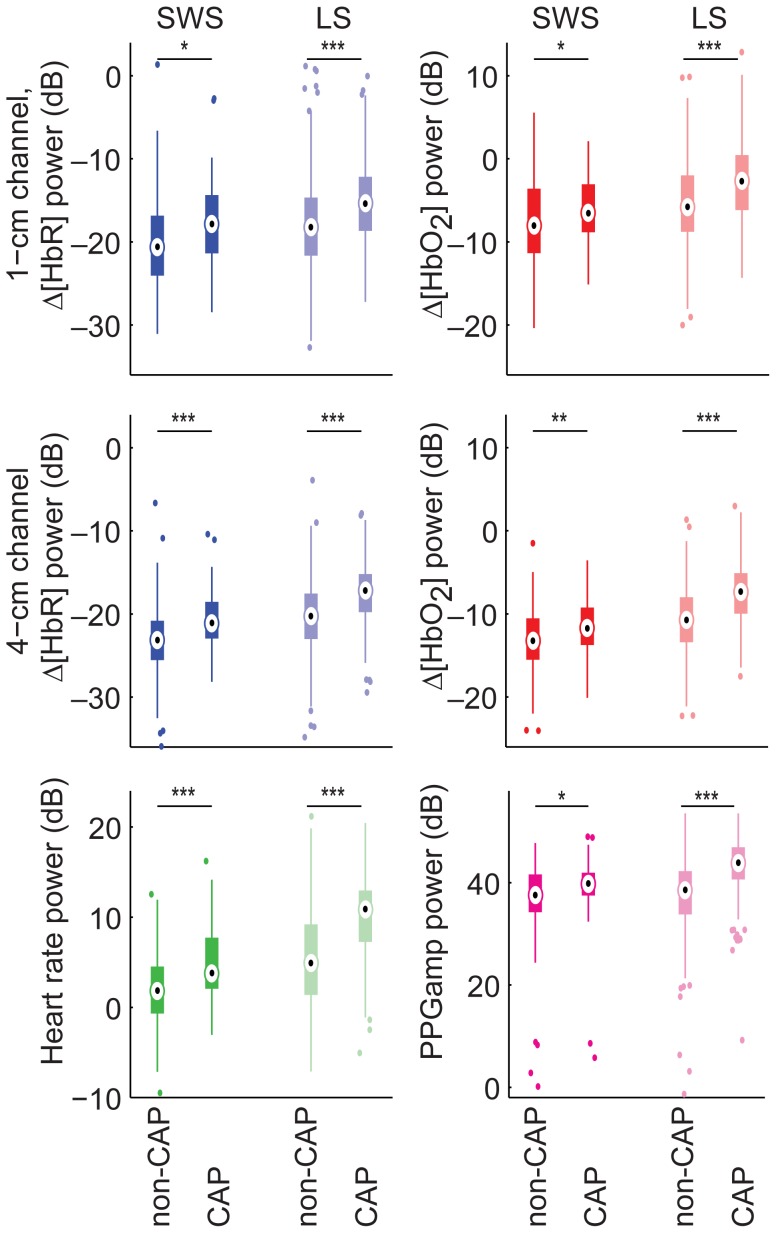
Power of VLF/LF oscillations in CAP versus non-CAP separately for SWS and LS. Statistically significant differences are marked with *p<0.05, **p<0.01, ***p<0.001 (*t*-test, *p*-values corrected with FDR control). The power of VLF/LF oscillations is higher during CAP than during non-CAP.

Alternating A and B produced oscillations in the hemodynamic data corresponding roughly to the repetition rate of the cycles (Figure 2B). The average repetition rate of all the cycles was 0.04±0.02 Hz. In all hemodynamic signals and both sleep stages, the power of VLF/LF oscillations was higher during CAP than during non-CAP (Figure 3).

**Figure 4:**
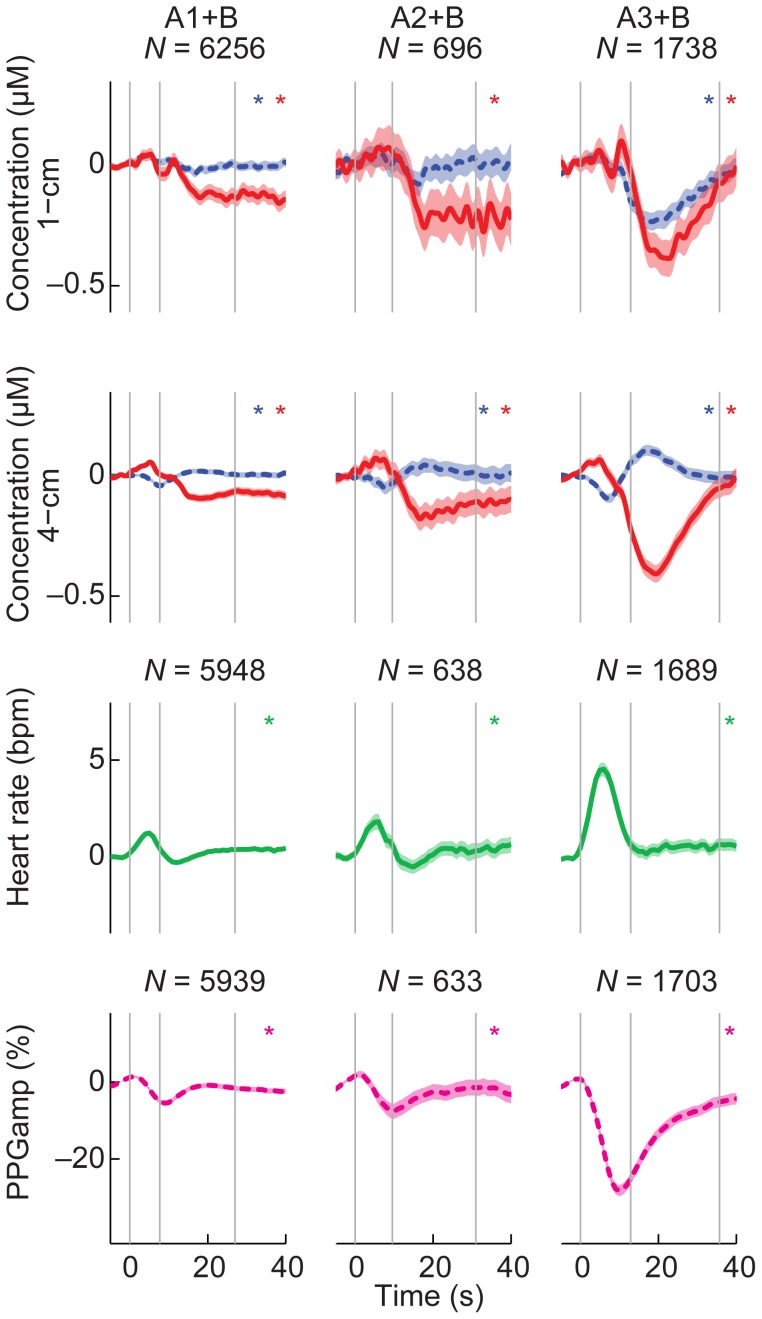
Averaged NIRS signals and cardiovascular signals in CAP subtypes. Rows represent individual NIRS channels and cardiovascular signals and columns separate CAP subtypes. Red solid line is Δ[HbO_2_] and blue dotted Δ[HbR] multiplied by two to show changes in the same scale. The shading depicts the 95% confidence interval of the mean and asterisks mark signals with significant changes during the cycle (one-way ANOVA with FDR-adjusted *p*-values). The vertical gray lines indicate the start of phase A, the average start of B and the average end of B. *N* indicates the number of cycles that have been averaged together and is same for both NIRS channels and signals. The cycle A3+B produces the strongest cortical and systemic hemodynamic changes. Changes in Δ[HbR] differ between the 1-cm and 4-cm channels.

Averaging hemodynamic signals over all CAP cycles (Figure 4) confirmed our observations from the unaveraged data: Δ[HbO_2_] showed a positive peak during A and a negative stronger peak during B. Heart rate increased and decreased back to the baseline during A. PPGamp peaked later than heart rate, decreasing during A and increasing slowly back to baseline during B. The behavior of Δ[HbR] differed between channels: it showed a negative peak in the 1-cm channel and a positive peak in the 4-cm channel during B. The strongest changes in all signals occurred during A3+B. SpO_2_ did not change during the CAP cycles; it was 97±1% in all subtypes during both A and B.

Statistical testing (Table 1) showed that Δ[HbO_2_], heart rate and PPGamp peak amplitudes differed between the subtypes of CAP (*p*<10^−3^). Post-hoc tests revealed that for all of these signals, subtype A3+B was stronger in magnitude than A1+B and A2+B. Δ[HbR] did not show main effects related to the CAP subtype because the peak amplitudes were negative in the 1-cm and positive in the 4-cm channel (*p*<10^−5^). However, the interaction between the NIRS channel and subtype of CAP was significant (*p* = 0.003). Post-hoc testing revealed that in both channels the magnitude of A3+B was stronger than that of A1+B and A2+B.

**Table 1:** Average peak times of hemodynamic signals, *p*-values of ANOVAs for peak amplitudes (only significant factors and interactions included), and results of post-hoc tests inside brackets.

	Δ[HbR]	Δ[HbO_2_]	Heart rate	PPGamp
**Peak time**	17 s	20 s	5 s	10 s
**CAP**	–	*p*<10^−3^ (A3*<A2*, A1*)	*p*<10^−8^ (A3>A2, A1)	*p*<10^−8^ (A3*<A2*, A1*)
**Duration**	–	*p* = 0.027 (L*<S*)	–	*p* = 0.018 (L*, M*<S*)
**Ch**	*p*<10^−5^ (1 cm*<4 cm)	–	N/A	N/A
**Cap×Ch**	*p* = 0.003 (1 cm *p*<10^−35^: A3*<A2*, A1*; 4 cm *p*<10^−14^: A3>A1, A2)	–	N/A	N/A

–: Not significant.

CAP: CAP subtype (A1, A2, A3).

Duration: Phase A duration (S = short 2–7 s; M = intermediate 7–11 s; L = long 11–60 s).

Ch: NIRS channel (1 cm, 4 cm).

*X*×*Y*: interaction between *X* and *Y*.

*X*<*Y*: amplitude in *X* is smaller than in *Y*.

* : negative amplitude; *X* ^*^<*Y* ^*^ means that *X* is stronger in magnitude than *Y*.

In addition to the CAP subtype, also the duration of phase A affected the peak amplitudes of Δ[HbO_2_] (*p* = 0.026) and PPGamp (*p* = 0.018). Short phases were smaller in magnitude than long ones (Δ[HbO_2_]) or smaller than long and intermediate phases (PPGamp).

Besides the CAP subtype and phase A duration, also the effect of the underlying sleep stage on the responses was investigated with ANOVA. It showed no significant effect on the signals. However, it should be noted that the number of cycles A2+B and A3+B was low in SWS, which might reduce the power of the statistical test.

## Discussion

We recorded CAP-related cortical and systemic hemodynamic changes in healthy subjects. The neuronal CAP activity was regularly accompanied by cortical and systemic hemodynamic changes, stating the physiological importance of autonomic regulation in situations in which arousal might take place. The cortical blood oxygenation and volume (sum of Δ[HbO_2_] and Δ[HbR] [29]) increased in the prefrontal cortex during phases A, followed by a stronger decrease during B. Cycles A3+B were related to stronger hemodynamic changes than the two other subtypes. Furthermore, we showed that CAPs manifest as slow oscillations in the hemodynamic signals; since CAP is repetitive by nature and its cycles produce hemodynamic changes at semi-regular intervals, CAP causes the hemodynamic signals to oscillate in the VLF/LF band.

The time spent in different sleep stages was in line with normal sleep macrostructure, but the CAP rates were higher in our study (64±12%) than what has been reported for young adults (32%), suggesting that the quality of sleep was lowered in this study [7], [30]. This effect on sleep microstructure may be attributed to the unfamiliarity of the laboratory environment and limited range of movement due to the recording cables. In addition, the CAP scoring was done with high sensitivity, assigning many CAP phases to the data, and thus possibly increasing the CAP rate. The average CAP cycle duration in our measurement (29±16 s) corresponded approximately to the normal value for young adults (28.5 s), as well as the duration of A (9±6 s) and B (20±6 s) compared to young adults (10.8 and 17.7 s, respectively) [30]. A3 was longer in duration compared to the other subtypes, which has also been reported before [30].

The presence of extracerebral components usually complicates the interpretation of NIRS data. The measurement is done through the scalp, and therefore extracerebral changes contribute to all signals, including the ones in channels with a long source-to-detector separation [31]–[33]. In addition, skin blood flow has been reported to increase followed by a marked decrease in response to K-complexes related to arousals and CAP [34]. The current data, nevertheless, allow for inferring about cerebral changes, since the polarity of Δ[HbR] differs between the 1- and 4-cm channels. An increase in Δ[HbR] during B is uniquely present in the 4-cm channel, and thus it most likely arises in the cortex, which the 1-cm channel samples remarkably less than the 4-cm channel [31], [35], [36]. Inaccuracy of the optical pathlength could also produce a change in the polarity of Δ[HbR] between the channels [37]. This is, however, unlikely since the pathlength was estimated directly from the data separately for the two wavelengths which were selected to be optimal for minimizing the cross-talk between Δ[HbO_2_] and Δ[HbR][37].

Extracerebral contribution may partly account for the Δ[HbO_2_] decrease in the 4-cm channel, as the skin blood flow has been reported to follow the same pattern as the observed Δ[HbO_2_] in response to K-complexes [34]. Nevertheless, the magnitude of Δ[HbO_2_] does not change between the 1-cm and 4-cm channels, proposing a decrease also in cortical Δ[HbO_2_]. A moderate increase in Δ[HbR] and large decrease in Δ[HbO_2_] in the 4-cm channel would suggest a decrease in the cerebral blood volume, as their sum, which is proportional to blood volume in the measured tissue [29], decreases. Furthermore, an increase in Δ[HbR] and a decrease in Δ[HbO_2_] are consistent with a decrease in tissue oxygenation. With constant oxygenation, the changes would have the same polarity. A possible origin of these changes is a decreased cerebral blood flow, i.e., the opposite of a hemodynamic response to brain activity [38], [39]. Alternatively, the signals may be affected by an increase in cerebral oxygen consumption, although this would only account for the decrease in oxygenation and not the blood volume decrease.

The systemic response suggests a transient increase in heart rate during A, accompanied by peripheral vasoconstriction which gradually subsides during B as indicated by PPGamp. These changes imply an increase in blood pressure towards the end of A. The PPGamp time course is roughly similar to the Δ[HbO_2_] signals except for a 5–10-s time difference, suggesting a delayed vasoconstriction in the scalp and/or in the brain during B.

A similar blood oxygenation pattern to the ones recorded in this study has been previously observed during sleep apnea [40]. In the present study, however, subjects with possible sleep apnea were rejected from the analysis and the SpO_2_ values did not change during the cycles, suggesting that the changes in Δ[HbR] and Δ[HbO_2_] are more likely related to microarousal rather than apnea. Supporting this view, cerebral blood flow velocity and blood-oxygen-level-dependent signals have been shown to drop as a consequence of arousals, consistently with an increase in local Δ[HbR] [8], [9]. The systemic and scalp hemodynamic changes also correspond to changes observed during microarousals [8], [10], [34], [41]–[44]. In addition, the NIRS changes presented in this study correspond to arousal- and CAP-related NIRS signals recorded during periodic limb movements [5], suggesting the CAP-related NIRS signals to be independent of the cause of the microarousal. The changes during phase A are comparable to changes observed in NIRS data during awakening or shifting from SWS to LS [13]. However, changes during B cannot be directly related to signal changes during falling asleep [13].

Reduced cerebral blood flow and oxygenation in A3+B expose the brain to transient hypoxia. The CAP rate, especially for the subtype A3, is increased in patients with some types of sleep disorders, e.g., insomnia or periodic limb movements [7], [45], [46]. These patients also have an increased risk for cardiovascular diseases [15], [47], which might be explained by their increased CAP rate (especially A3): the intermittent cerebral hypoxia may cause harmful reactions in the cardiac and brain blood vessel walls [48]. However, since the applicability of NIRS for quantitative estimation of hemoglobin concentration changes is limited due to the partial volume effect [49], it is difficult to assess the severity of the transient hypoxia.

Recurring CAP cycles cause the hemodynamic signals to oscillate, as shown in this study by the increased power of VLF/LF oscillations in CAP compared to non-CAP. Instead of being centered around one frequency, the oscillations occur within a range that is limited by the repetition rate of the cycles, i.e., from 0.008 to 0.25 Hz. In addition, the CAP sequences themselves are modulated at even lower frequencies (CAP sequence on – CAP sequence off). All of these frequencies lie within the VLF/LF band, which has been reported to have reduced amplitude in SWS compared to LS [13]. A part of this reduction may be attributed to the lowered A3+B activity in SWS: cycles A3+B produce the strongest hemodynamic changes and the time spent in these cycles is reduced in SWS (3%) as compared with LS (20%).

These slow cortical hemodynamic oscillations related to CAP may also be coupled to infraslow electric oscillations (0.02…0.2 Hz) modulating the cortical excitability, since they may drive CAP sequences at least partly [50]. The EEG recording in this study had the bandwidth 0.5…90 Hz; thus, we could not evaluate the relation between the infraslow oscillations and hemodynamic oscillations.

## Conclusions

Circulation must be able to react rapidly if sleep is abruptly interrupted. A partial activation of arousal mechanisms in the brain during sleep, as indicated by CAP in EEG, is related to both cortical and systemic hemodynamic changes, demonstrating the importance of autonomic regulation in situations with a potential for arousal. Hemodynamics during CAP indicates a transient cortical hypoxia during phases B, which may be one of the factors mediating the harmful effect of unstable sleep on the cerebral vasculature and neuronal function.
